# Assessing professional equipoise and views about a future clinical trial of invasive urodynamics prior to surgery for stress urinary incontinence in women: A survey within a mixed methods feasibility study

**DOI:** 10.1002/nau.22328

**Published:** 2012-09-28

**Authors:** Paul Hilton, Andy Bryant, Denise Howel, Elaine McColl, Brian S Buckley, Malcolm Lucas, Douglas G Tincello, Natalie Armstrong

**Affiliations:** 1 Directorate of Women's Services, Royal Victoria InfirmaryNewcastle upon Tyne, UK; 2 Institute of Health and Society, Newcastle UniversityNewcastle upon Tyne, UK; 3 Clinical Trials Unit, Newcastle UniversityNewcastle upon Tyne, UK; 4 Bladder & Bowel FoundationLondon, UK; 5 Department of Urology, Morriston HospitalSwansea, UK; 6 Department of Obstetrics and Gynaecology, University of LeicesterLeicester, UK; 7 Department of Health Sciences, University of LeicesterLeicester, UK

**Keywords:** healthcare surveys, interview studies, randomized controlled trial, urodynamics

## Abstract

**Aims:**

To determine surgeons' views on invasive urodynamic testing (IUT) prior to surgery for stress (SUI) or stress predominant mixed urinary incontinence (MUI).

**Methods:**

Members of British Society of Urogynaecology (BSUG) and British Association of Urological Surgeons Section of Female, Neurological and Urodynamic Urology (BAUS-SFNUU) were sent an email invitation to complete an online “*SurveyMonkey*®*”* questionnaire regarding their current use of IUT prior to surgical treatment of SUI, their view about the necessity for IUT in various clinical scenarios, and their willingness to randomize patients into a future trial of IUT. A purposive sample of respondents was invited for telephone interview to explore further how they use IUT to inform clinical decisions, and to contextualize questionnaire responses.

**Results:**

There were 176/517 (34%) responses, 106/332 (32%) from gynecologists/urogynecologists and 67/185 (36%) from urologists; all respondents had access to IUT, and 89% currently arrange IUT for most women with SUI or stress predominant MUI. For a variety of scenarios with increasingly complex symptoms the level of individual equipoise (“undecided” about IUT) was very low (1–6%) and community equipoise was, at best, 66:34 (IUT “essential” vs. “unnecessary”) even for the simplest scenario. Nevertheless, 70% rated the research question underlying the proposed studies “very important” or “extremely important;” 60% recorded a “willingness to randomize” score ≥8/10.

**Conclusions:**

Most urogynecologists and urologists consider IUT essential before surgery in SUI with or without other symptoms. Most however recognize the need for further research, and indicated a willingness to recruit into multicenter trials addressing this question. Neurourol. Urodynam. 31:?–?, 2012. © 2012 Wiley Periodicals, Inc.

## INTRODUCTION

Urinary incontinence (UI) whilst rarely life-threatening, may seriously influence the physical, psychological, and social wellbeing of affected individuals. Prevalence figures for UI range from 5% to 69% in women 15 years and older, with most studies in the range 25–45%; more severe UI is reported in 4–7% of women under 65 years, and around 5 million women over 20 years of age in England and Wales may be affected,^1^ of whom 65–85% have stress (SUI) or mixed incontinence (MUI).^2^

Several methods are used in the assessment of UI to guide management decisions. These include non-invasive tests (such as free urine flow rate and post-void residual volume), and invasive urodynamic tests (IUT), distinguished by the need for catheterization (such as conventional cystometry, videourodynamics, long-term ambulatory bladder pressure monitoring, urethral pressure measurements, and leak point pressures). The costing report associated with the National Institute for Health and Clinical Excellence (NICE) clinical guideline on UI calculated the annual national cost of urodynamic investigations in the UK to be over £22m in 2006.^3^

In a UK survey in 2002, only half of the units surveyed had guidelines on indications for the tests, and 85% carried out cystometry in all women with incontinence.^4^ Current guidance from NICE suggests that cystometry is not required prior to conservative treatments, and that, whilst it may be needed in more complex clinical scenarios, there is no evidence to support the use of IUT prior to surgery where the diagnosis of SUI is likely based on history alone.^5^

NICE,^5^ National Institute for Health Research-Health Technology Assessments (NIHR-HTA),^6^ the Cochrane Collaboration,^7^ and the International Consultations on Incontinence (ICI),8–11 have all undertaken systematic reviews on the subject of urodynamics, and all emphasize the lack of high quality primary research confirming clinical utility. The clinical usefulness of IUT was also among the top prioritized unanswered research questions identified within a research priorities setting exercise involving eight patient and 13 professional organisations.^12^, ^13^

There are several reasons to conduct a pilot trial and feasibility assessment before undertaking a definitive trial of IUT prior to surgery for SUI or stress predominant MUI. These were reviewed in the background to the published protocol for this study.^14^ The INVESTIGATE-I study was therefore planned as a mixed methods study looking at the feasibility of a definitive trial of the clinical and cost-effectiveness of IUT prior to surgery for SUI or stress predominant MUI.^15^ It consists of four elements:

(1) A pragmatic multicenter “rehearsal” pilot randomized controlled trial.
(2) Interviews with a purposively sampled subset of women, some of whom had agreed and some declined randomization within the pilot trial.
(3) National survey of clinicians' views on IUT.
(4) Qualitative interviews with a purposively sampled subset of those clinicians responding to the survey.

This article reports results from the last two of these elements. The aim of these was to determine the views of surgeons on the role of invasive urodynamic testing (IUT) prior to surgical treatment of stress (SUI) or stress predominant mixed urinary incontinence (MUI), and to establish their willingness to recruit patients into a future definitive trial.

## METHODS

Survey information materials and data collection instruments were approved by the Research Committees of the British Society of Urogynecology (BSUG) and the British Association of Urological Surgeons Section of Female, Neurological and Urodynamic Urology (BAUS-SFNUU). Initial draft versions of the survey materials had previously been piloted for content validity and functionality of the online system by a small group of gynecologists and urologists, who were not BSUG or BAUS-SFNUU members.

The organizations circulated study information and invitations to participate to their respective memberships. An introductory email was sent that included: a description of the INVESTIGATE studies; links to further information on the NIHR-HTA (http://www.hta.ac.uk/project/2272.asp) and trial (http://www.investigate-trial.com) websites; a link to the “*SurveyMonkey*®” site where the questionnaire was hosted; contact details should potential respondents prefer to obtain a paper-based questionnaire and reply-paid envelope. A copy of the paper-based questionnaire is included as Appendix 1.

The survey content itself collected categorized demographic data regarding respondents' grade or rank, role (specialty and extent of specialization), gender, time since graduation, access to and current use of IUT, and their current workload in surgery for SUI. In order to assess current use of urodynamics in the patient group of interest, we asked respondents “Do you currently arrange invasive urodynamic tests for most (say 75%) of your female patients with stress or stress predominant mixed incontinence?”

Respondents were presented with the clinical scenario of a 45-year-old woman with two children, who has been sterilized; she had previously undergone pelvic floor muscle training and possibly other conservative treatments, without benefit; she had not had any previous continence surgery. They were then given six urinary symptom descriptions of varying complexity (see Table II). Using a modified version of a bidirectional scale developed for measuring clinician and patient preferences in surgery,^16^ they were asked to rate the strength of their views about the necessity for IUT before undertaking surgical treatment on a 11-point Likert scale from “unnecessary” (5) through “undecided” (0) to “essential” (−5; see Table II). They were specifically asked to respond on the basis of their own opinion, regardless of their current practices, and regardless of what they might have read in recent literature or current guidelines.

Respondents were asked to use a Likert-type scale graded “not at all important,” “somewhat important,” “very important,” or “extremely important” to express their views about the importance of the research question, “Does *invasive* urodynamic testing prior to surgical treatment of stress or stress predominant mixed urinary incontinence improve the clinical- and cost-effectiveness of treatment compared to clinical assessment with *non-invasive testing*?” A vignette of the design of a proposed definitive trial was presented, and respondents asked their willingness to participate and to randomize patients within such a trial. For those unwilling to randomize, open questions with free text responses asked about their reasons for their view, and about acceptable alternative trial designs. Finally, the questionnaire asked about respondents' willingness to participate in a short telephone interview to explore further how they use the results of urodynamic investigations to inform their clinical decisions, and to contextualize the questionnaire responses; if willing, respondents were asked to provide preferred contact details and optimum time for contact.

In order to maintain confidentiality of email addresses, the invitation email and “*SurveyMonkey*” link were sent out to their respective members by the BSUG and BAUS-SFNUU secretariats in August 2011. Reminder emails were sent at 3 and 6 weeks after the initial circulation to all potential respondents, as it was not possible to target the reminders to non-respondents. The survey site was closed 12 weeks after the initial invitations. There are very few individuals who are members of both organizations, but a footnote was appended to the letter apologizing for dual circulation, and requesting that they make only a single response.

### Interview Study

A purposive sub-sample was drawn from those respondents indicating a willingness to take part in the interview study, and who provided contact details. Interviews continued until a point of saturation was reached (i.e., that no new material was emerging from the interviews). Telephone interviews were undertaken by an experienced qualitative researcher using a topic guide based on the survey and developed through discussion within the project team. The topic guide ensured all areas of interest were covered, but was used flexibly with the aim of allowing interviews to flow as freely and naturally as possible and to allow participants to discuss issues that were important to them. The interviewer prompted as appropriate to ensure that all views were fully explained, and the meaning of participants' responses clear. All interviews were audio-recorded and transcribed verbatim.

### Statistical Analysis

The analysis was carried out on the complete dataset following closure at 12 weeks after initial circulation. Basic descriptive statistics including response rates, percentages in categories and summary statistics were used for all relevant outcomes. No attempt was made to impute missing data for any of the outcomes. “Equipoise ratios” were calculated after Young et al.^16^ to report the three proportions for each scenario: those clinicians who regarded IUT as essential (to a greater or lesser extent) that is, gave scores of −5 to −1, had no preference between using IUT or not that is, gave a score of 0, and those who regarded it as unnecessary (to a greater or lesser extent) that is, gave scores of +1 to +5.

### Qualitative Analysis

Analysis of the interview data was based on the constant comparative method.^17^ Transcripts were read three to four times and open codes initially applied line-by-line to the data to represent the meaning or significance of each sentence or group of sentences. Generation of the open codes proceeded sequentially, with no attempt at this stage to impose any framework on the data. The open codes were then incrementally grouped into organizing categories or themes. These categories were modified and checked constantly as further open codes were incorporated as analysis proceeded. When categories had been created to express all of the open codes, explicit specifications were written for each of the categories to assist in determining under what circumstances data should be assigned to any given category. The categories and their specifications (the coding scheme) were then programmed into the NVivo qualitative software. The coding scheme was then used to process the dataset systematically by assigning each section of text to a category, according to the category specifications.

## RESULTS

### Responses

The BSUG and BAUS-SFNUU membership databases are fluid, with new members joining and others leaving continuously throughout the year; hence the numbers sent reminder letters were slightly different from initial invitations. Initial invitations went to 332 BSUG members and 185 BAUS-SFNUU members, with most of these, plus a small number of new members, being sent reminder emails to follow up the initial invitation. A total of 176/517 (34%) responded to the clinician survey, with the majority answering most of the questionnaire.

Of those responding, 55% did so after the initial circulation, 36% after the first reminder letter, and 9% after the second reminder. Following each circulation, the majority of responses were received within the first week (97%, 63%, and 100%, respectively), with 97%, 79%, and 100% being within 2 weeks.

### Demographics

Table I provides baseline characteristics of those who responded to the survey. The specialist societies were able to provide only limited information about the demographic of their respective membership. Of the 332 BSUG members, 76% were full (consultant) members, 23% associate (non-consultant) members, and 1% emeritus (retired) members. BAUS-SFNUU had 185 full members who were all consultants.

**Table I:** Demographic Variables Amongst Responders

Explanatory variable	*n* (N = 176)	%
Current grade/rank		
Trainee	10	5.7
Specialty doctor	8	4.5
Consultant	158	89.8
Current clinical role		
Generalist	6	3.4
Special Interest	115	65.3
Subspecialist	55	31.3
Specialty		
Gynecologist	106	60.2
Urologist	67	38.1
Other	3	1.7
Gender		
Male	117	66.5
Female	59	33.5
Years since graduation from medical school		
0–5 years	1	0.6
6–10 years	6	3.4
11–15 years	17	9.7
16–20 years	40	22.7
21–30 years	80	45.5
31–40 years	32	18.2
Undertake urodynamic investigations		
Yes	141	80.1
No	35	19.9
Access to or undertake urodynamics		
No	0	0.0
Yes	176	100.0
Volume of SUI operations per year		
0–10	18	10.2
11–50	96	54.5
51–100	46	26.1
101–200	15	8.5
More than 200	1	0.3
Arrange cystometry for >75% of patients		
Yes	157	89.2
No	19	10.8

The response rates were similar between specialities (BSUG 32.9%; BAUS-SFNUU 36.2%); amongst BSUG members, consultants were more likely to respond than non-consultants and amongst BAUS members women were more likely to respond than men.

### Urodynamic Access and Use

All respondents reported having access to urodynamic facilities for their patients, and 80% undertook urodynamic investigations themselves; 89% indicated that they currently arrange IUT in most of their female patients with SUI or stress predominant MUI.

### Clinical Scenarios

Responses in terms of the necessity for IUT in the six clinical scenarios are given in Table II. For each of these scenarios, only 1–6% of respondents were undecided, with most reporting highly polarized opinions that is, towards the left or right ends of the Likert scale. For the three more complex symptom descriptions “equipoise ratios” averaged 93:3:4. For the three simpler symptom descriptions, which might be summarized as “stress or stress predominant mixed incontinence,” and comprise the patients intended as eligible in the pilot and future definitive trials, a greater range of opinions was expressed. However, even in scenario 1 (pure stress incontinence that is clinically demonstrable), two-thirds thought it necessary to a greater or lesser extent (i.e., gave scores of −1 to −5), with over a third of respondents considered IUT essential (i.e., gave a score of −5): the “equipoise ratio” was 66:1:33.

**Table II:** Responses in Terms of the Necessity for IUT in the Six Clinical Scenarios (Given as % of n Responses for Each Point on Scale, and Summary “Equipoise Ratio” = Sum to Left: Undecided: Sum to Right)

11-point scale	Essential						Undecided	Unnecessary											
	−5	−4	−3	−2	−1	0	1	2	3	4	5
Scenario 1 (n = 172)	Complains of stress incontinence, but no frequency, nocturia, urgency or urgency incontinence; no difficulty; stress incontinence on clinical examination. Pure SUI; stress leak IS demonstrable											
Opinion (%)	37	11	9	6	3	1	1	3	5	2	23
“Equipoise ratio”	66%						1%	34%											
Scenario 2 (n = 172)	Complains of stress incontinence, but no frequency, nocturia, urgency, or urgency incontinence; no difficulty; stress incontinence demonstrated on clinical examination. Pure SUI; stress leak NOT demonstrable											
Opinion (%)	61	14	5	3	1	6	1	3	3	1	4
“Equipoise ratio”	83%						6%	12%											
Scenario 3 (n = 170)	Complains of stress incontinence, mild frequency, urgency and urgency incontinence, but describes more significant problem; no voiding difficulty. STRESS predominant mixed UI											
Opinion (%)	73	7.5	6	2	0.5	5	1	2	0.5	0.5	2
“Equipoise ratio”	89%						5%	6%											
Scenario 4 (n = 171)	Complains of stress incontinence, frequency x10, nocturia x2, urgency and urgency incontinence, of similar magnitude; no sympt difficulty. EQUAL severity mixed UI											
Opinion (%)	86.5	4	3	2	0	2	0.5	0	0	0	2
“Equipoise ratio”	96%						2%	3%											
Scenario 5 (n = 172)	Complains of stress incontinence, frequency x15, nocturia x2, urgency and urgency incontinence, urge as the more significant pro symptoms of voiding difficulty. URGE predominant mixed UI											
Opinion (%)	85	3.5	2.5	0.5	0.5	2	0.5	1	1	0	3.5
“Equipoise ratio”	92%						2%	6%											
Scenario 6 (n = 172)	Complains of stress incontinence, but no frequency, nocturia, urgency or urgency incontinence; also poor flow, and feeling of inco emptying. Pure SUI; symptoms of VOIDING difficulty											
Opinion (%)	84.5	3.5	3	1.75	0	4	1.75	0	0.5	0	1
“Equipoise ratio”	93%						4%	3%											

### Views About a Future Definitive Trial

These results could be interpreted to suggest that clinicians had little doubt about the value of IUT and would therefore not be interested in a future clinical trial. However, when asked to rate the importance of the research question, overall 24% rated it “extremely important,” 45.5% “very important,” 26% “somewhat important,” and only 4.5% thought it “not at all important.”

**Figure 1:**
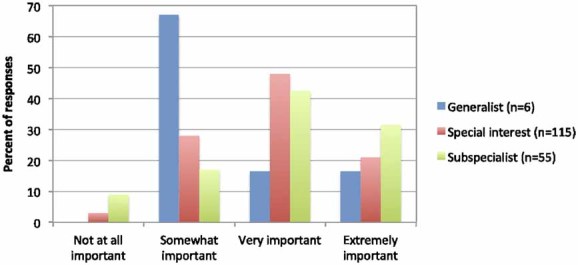
Responses by level of specialisation in relation to the importance of the research question.

Responses did not differ markedly between the main specialties, although generalists were somewhat less likely to look on the question as being “extremely important” or “very important” (33%), than consultants with a special interest (69%), or subspecialists (74%; see Fig. .1).

**Figure 2:**
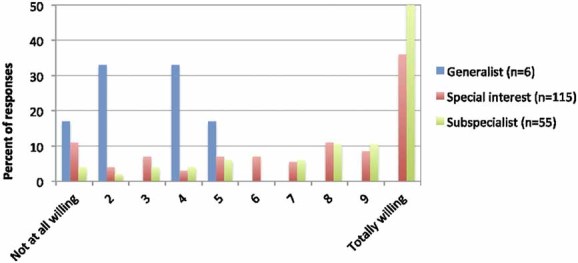
Likert scale of ‘willingness to randomise’ by level of specialisation.

On the 10-point Likert scale of “willingness to randomize” overall, 60% gave a score of eight or over. Again, the generalist consultants were less likely to indicate willingness to recruit into a trial (0% indicated a level of ≥8) than consultants with a special interest (56%) or subspecialists (74%; see Fig. .2).

### Interview Study

Of the 176 respondents, 87 (49%) agreed to being approached for interview and provided contact details. A diverse sample was recruited purposively including: gynecologists and urologists; those who did/did not routinely use IUT; those with different approaches to when IUT was needed; those with different perspectives on both the planned RCT and their willingness to randomize patients. A total of 18 interviews were carried out.

As would be expected from the quantitative results, interview participants tended to be polarized in their view and regard IUT as either essential or as of limited use. For those interviewees who undertook IUT regularly, the tests seemed to have a range of functions that clinicians regarded as valuable. These included: adding to the overall picture of what's going on and helping to inform the best course of action; acting as a “safety net” to prevent unnecessary or inappropriate surgery; and facilitating appropriate counseling of patients."I would use urodynamic tests on anyone that I was going to operate on, it's very easy to operate, but it's not very easy to un-operate, so if you have a complication that arises as a result of your surgery, you can't go back and say “well I would have liked that information, if I'd known that beforehand, I would have done something different" (Participant 02).

Interestingly, there was also an element of fitting in with what colleagues did, and thereby adopting local customs and practices. I have just moved to a new Trust, my colleague investigates all patients who have stress incontinence before surgery. In my previous post I didn't actually undertake urodynamics in patients who had pure stress incontinence symptoms so at the moment I, I suppose you could say that I'm doing it because it's, it's sort of departmental protocol really (Participant 09).

For those interviewees who used IUT relatively rarely, this position was underpinned by a range of factors including: the perception that the use of IUT is not currently recommended by NICE; perceived unnecessary time and cost implications; a perception that IUT would not alter the treatment plan; and a belief that the information that could be obtained from other sources (such as patient history, examination, and bladder diary) was sufficient. "We have things like flow meters which, you know, in the clinic, we have bladder scans, we can measure residuals, patients are quite good at filling in frequency volume chart […] and a good physical examination combined with these non-invasive tests that I've just mentioned, I think gives you much more information than urodynamics (Participant 01).

As in the overall survey responses, about two thirds of the clinicians interviewed thought the basic research question of the INVESTIGATE studies to be an important one. For some, this was because they believed there was genuine uncertainty about the benefit of IUT. I think it is worth doing because as well as telling us whether urodynamics is useful, there will be a lot of information which will tell us in what ways it can be useful, it will say these are the things you should be looking at (Participant 16).

However, for others, this was because they believed they knew the answer to the question but felt the need for “harder evidence” to back them up and encourage others to change their practice. Within the sample, there were examples both of interviewees who believed a trial would show IUT should be used, and those who believed a trial would show the opposite. I still think it is important that we answer this question because you know my certainty up to now is based on what I have been taught and what I have observed but that is not based on research particularly so I still think it is a very important research question (Participant 17).

WHEN WE ASKED YOU WHETHER OR NOT YOU THOUGHT THE QUESTION THAT INVESTIGATE IS ADDRESSING IS IMPORTANT, YOU SAID VERY IMPORTANT.

Well on the basis of what the NICE guidelines said if we stopped doing it in the large number of cases that they suggest we should stop, then it would free up funding for something else (Participant 08).

As a deliberate outcome of the purposive sampling, we interviewed fairly even numbers of both those who would be willing to randomize into a definitive trial and those who would not. Unsurprisingly, those who always undertook IUT and regarded it as essential would often not be willing to randomize, even if they had indicated they thought it an important research question. In these cases they wanted the question answered in order to provide hard evidence that IUT is necessary, but they were not prepared to allow their patients to be part of producing that evidence. I wouldn't be happy [to randomise patients], no. That's in keeping with my belief that it is an important test (Participant 12).

Those who appeared genuinely uncertain about IUT, or were happy not doing it, were the ones that seemed happiest to randomize.

## DISCUSSION

In this study, directed at identifying professional opinion within a specialist group interested in the management of female urinary incontinence, we have shown that the majority of BAUS-SFNUU & BSUG members who responded to the survey consider IUT to be necessary to a greater or lesser degree before surgical intervention in SUI, whether or not patients have additional symptoms suggestive of overactive bladder or voiding dysfunction. Not only are few clinicians apparently undecided on the issue (i.e., are in personal equipoise), but there is little evidence of professional community equipoise. Nevertheless, many see the need for more research on this question, and indicated their willingness to randomize patients into trials seeking to investigate the clinical utility of IUT. The results of our interview study give some insights into this apparent inconsistency.

The overall response rate to the questionnaire (34%) must leave any conclusions open to question, due to the potential for non-response bias. It is possible that those who did respond may hold systematically different views on the use of IUT and on the research question than those who did not participate in the survey, but this cannot be confirmed. A number of previous surveys of similar national and international professional groups have been published, with response rates between 21% and 67%.18–21 None of these studies employed incentives to take part, and indeed none used reminder letters or emails.^22^, ^23^ Clearly the level of interest or excitement generated by the topic in potential respondents is of importance in encouraging responses. It is however difficult to explain why surveys on such similar topics should achieve such varying response rates in different countries (21–57%)^18^, ^20^ or why a survey on a clinical guideline^19^ should achieve a different response rate from one on a major recommendation from the same guidance (this study) (64% vs. 34%).

Whilst the majority of responses came in after the initial circulation (55%), a significant proportion followed the first and second reminders (36% and 9%, respectively). This is in line with published data demonstrating the benefit of reminders.^22^, ^23^ Following each of the three circulations the majority of responses were obtained within one (mean, 87%) or two (mean, 92%) weeks. The rate of responses suggests that we might have achieved a similar outcome by shortening the time between circulations to 2 weeks, and closing the survey site at 7 rather than 12 weeks.

The limited information on the specialty group membership makes comparison of respondents and non-respondents difficult. The similar rates of response by BSUG and BAUS-SFNUU members, and the higher response rate amongst consultants than non-consultants suggest that the responses are likely to reflect relevant surgical opinion.

Only when clinicians are in equipoise on an issue or recognize it to be an area of genuine uncertainty are they likely to feel comfortable to randomize their patients between treatments or, as in this case, investigation strategies. Hence, measuring surgeon preference is a crucial component of trial feasibility. In a survey of clinician opinion about the feasibility of a planned trial of surgery for incontinence and pelvic organ prolapse, whilst a majority of respondents thought the study ethical, only a third would agree to randomize patients.^24^

From the point of view of the individual respondent the position of equipoise would be indicated by an “undecided” response. With true equipoise within the professional community one might similarly expect most or all responses to be in the “undecided” category, and few either to the left (“essential”) or right (“unnecessary”). However community equipoise could also be said to exist when there is a balance of opinion across the spectrum of responses that is, with a similar number to left and right of the “undecided” position. The “equipoise ratios” demonstrated that very few respondents saw themselves as being undecided on the issue of IUT in any of the clinical scenarios, with the majority in each case feeling that investigation was essential before undertaking surgical treatment. Only in the context of “SUI or stress predominant MUI” was there any indication of a spread of opinion, and only in “pure SUI that is clinically demonstrable” was there a significant number of respondents who would consider IUT to be unnecessary.

Despite this lack of personal equipoise, and the fact that IUT was considered necessary across all scenarios, the majority of respondents regarded the basic research question as being important (70%), and most would be prepared to randomize patients into a definitive RCT to address this (60%). Analysis of the interview study data gives some insight into the reasons for this apparent inconsistency. It might be anticipated that clinicians would only regard a research question as important, and be prepared to randomize their patients in a study, in cases where they themselves were uncertain of the best course of action and looking to the study as a means of resolving that uncertainty. However, discussion of these issues in interview revealed a more complex picture. While some clinicians' views were shaped by genuine uncertainty about the value of IUT, more commonly the research question was regarded as important because clinicians believed they knew the answer and wanted research in order to change others' practice and bring it in line with their own. This could introduce an important complicating factor to whether they would be prepared to randomize patients because clinicians who regarded IUT as essential may not be willing to have some of their patients be denied it. While recognition of a degree of community equipoise may allow many to “suspend” their lack of personal equipoise and agree to randomize their patients in to a future definitive trial,^25^ it is likely that some will find this unacceptable.

A decision analysis study from the USA failed to find support for IUT before surgery in women likely to have SUI.^26^ A similar economic assessment within the NICE report on UI, using assumptions more applicable to current NHS practice found that for every 10,000 patients assessed there would be approximately 13 additional cures using IUT, at an additional cost per cure of £26,125. With a “willingness to pay” threshold of £20,000 per quality-adjusted life year (QALY)^27^ each cure would have to generate 1.3 QALYs for invasive urodynamics to be considered cost effective;^5^ a recent UK randomized trial found that surgery for SUI generated only 0.8 QALYs per procedure, suggesting that preoperative IUT would not be cost-effective compared to no additional investigations.^28^

One small RCT showed no significant benefit from IUT prior to conservative treatment, although this had methodological issues confounding interpretation.^29^ In a cohort study from the North Thames region, women were no more likely to benefit from incontinence surgery if they had undergone preoperative IUT,^30^ and a US study of Medicare patients found that those who had preoperative IUT appeared more likely to develop urge incontinence after their surgery.^31^ Secondary analyses of data from a US randomized controlled trial found that preoperative IUT did not predict failure or postoperative voiding dysfunction,^32^ and that the presence of detrusor overactivity preoperatively did not predict success in women with pure or predominant SUI.^33^ Clearly these conflicting data emphasize the need for large robust randomized trials in this area.

Whilst undertaking the INVESTIGATE-I study we became aware of two on-going definitive trials addressing similar research questions; these have since been published as the ValUE study from the USA,^34^ and the VUSIS from Netherlands.^35^ Both used a non-inferiority design, and both found basic clinical assessment to be non-inferior to IUT. Both studies considered women with SUI or stress predominant MUI, although in contrast to our own studies, only those with clinically demonstrable stress leakage were included. The VUSIS study was terminated prematurely in view of slow recruitment, failed to achieve its planned sample size; the validity and reliability of its conclusions could therefore be questioned. The ValUE study was powered with a non-inferiority margin of 11% which we consider somewhat high and a difference that might potentially influence the decisions of both clinicians and patients. We acknowledge that the latter trial may be considered to provide an answer to our research question, and that, were it available at the time our survey was distributed, it may have influenced the response of some surgeons. Nevertheless, we feel there is still a need to confirm these findings in a different healthcare setting, and to establish not only clinical but also cost-effectiveness comparisons.

## CONCLUSIONS

The majority of BAUS-SFNUU & BSUG members who responded to this survey consider IUT to be essential before surgical intervention in SUI with or without other symptoms. Most however recognize a gap in our knowledge, best addressed by a large pragmatic multicenter RCT, and most indicate their willingness to randomize patients into a trial.

Being in equipoise over a research question that is, recognizing it to be not only an important issue, but also an area of uncertainty, is generally seen to be a pre-requisite for those recruiting patients into a randomized trials. Reconciling the lack of individual equipoise with the principle of random allocation of investigation strategy presents a challenge to the implementation of a future definitive RCT in UK; the recognition of professional community equipoise however may facilitate this.

Our definition of feasibility, and hence our decision whether or not to proceed with a definitive trial, is a combined function of the outcomes of the various elements of the INVESTIGATE-I study. This includes: the confirmation that units are able to identify eligible women and to recruit and randomize them; the acceptability of the investigation strategies as manifest though recruitment and retention levels; the feasibility and acceptability of data collection tools measured by completion rates and quality of data; and clinical outcome data to estimate the necessary sample size. The majority of these data will come from the results of the pilot trial, and qualitative patient interviews. The results of this survey and the qualitative interviews with surgeons, give additional insight into the numbers and types of clinicians and units that may wish to contribute to a definitive study, and may usefully inform the content of future study materials.

## 

Conflict of interest: none.

Ethical Approval: Favorable ethical opinion was received from Newcastle and North Tyneside No.1 Research Ethics committee on 06/01/2011 (their minute reference: 10/H0906/76).

P.H.: No current financial interests; previous chair of NICE Guideline Development Group (GDG) on urinary incontinence (UI) in women; previous member NETSCC-HTA Interventional Procedures Panel, and Clinical Evaluations and Trials Prioritization Group; previous commercial research funding for trials of surgery for stress incontinence from *Gynecare* (1998–2003) and *Gyne Ideas* (now *Mpathy Medical*; 2001–2003). M.L.: No current financial interests; previous member of NICE GDG on UI in women and on lower urinary tract symptoms in men; chair European Association of Urology guideline group on UI. D.G.T.: Consultancy work for *Pfizer*, *Ethicon*, *Galen*; research grants from *Ethicon*, *Astellas*. Chair of BSUG Research Committee. Executive Editor of BJOG, Vice-chairman of the Wellbeing of Women Research Advisory Committee. A.B., D.H., E.M., B.B., N.A.: None.

Author's Contributions: P.H. is the lead grant holder, he conceived the study, led on the protocol development, questionnaire design and writing the manuscript, and approved the final version for publication. D.H., E.M., B.B., M.L., D.G.T., N.A. are co-holders of the grant, contributed to protocol development and to writing the manuscript, and approved the final version for publication. E.M.: additionally led on questionnaire formatting. A.B., D.H.: additionally undertook the statistical analysis. N.A.: additionally led on the interview study.
