# Supplementary figures and images for: Assessing professional equipoise and views about a future clinical trial of invasive urodynamics prior to surgery for stress urinary incontinence in women: A survey within a mixed methods feasibility study
Source: Neurourol Urodyn. 2012 Sep 28;31(8):1223–30. doi: 10.1002/nau.22328 (PMC3504983; doi:10.1002/nau.22328)

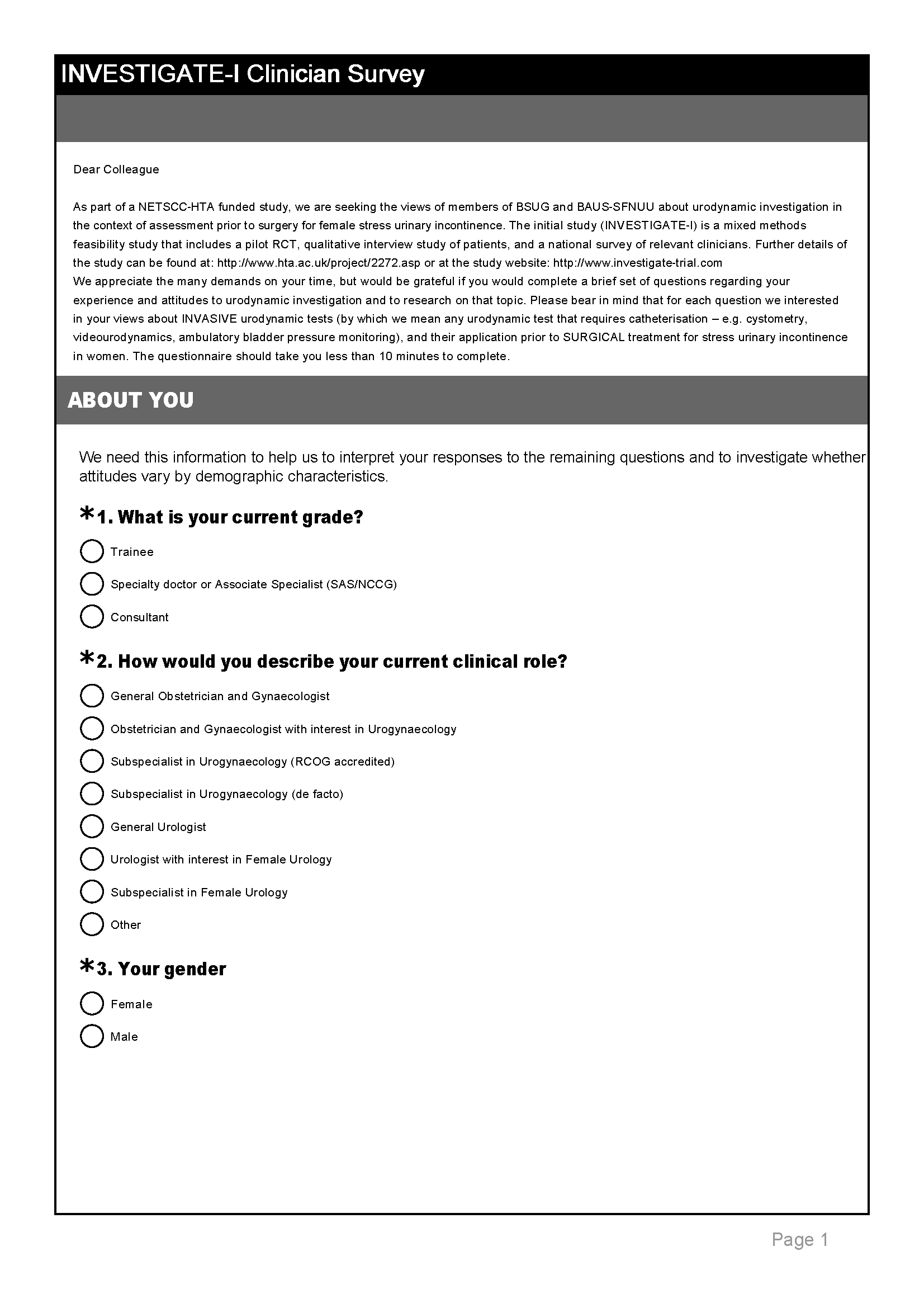

Supplement: Supplementary file 1 [file nau0031-1223-sd1.tif]

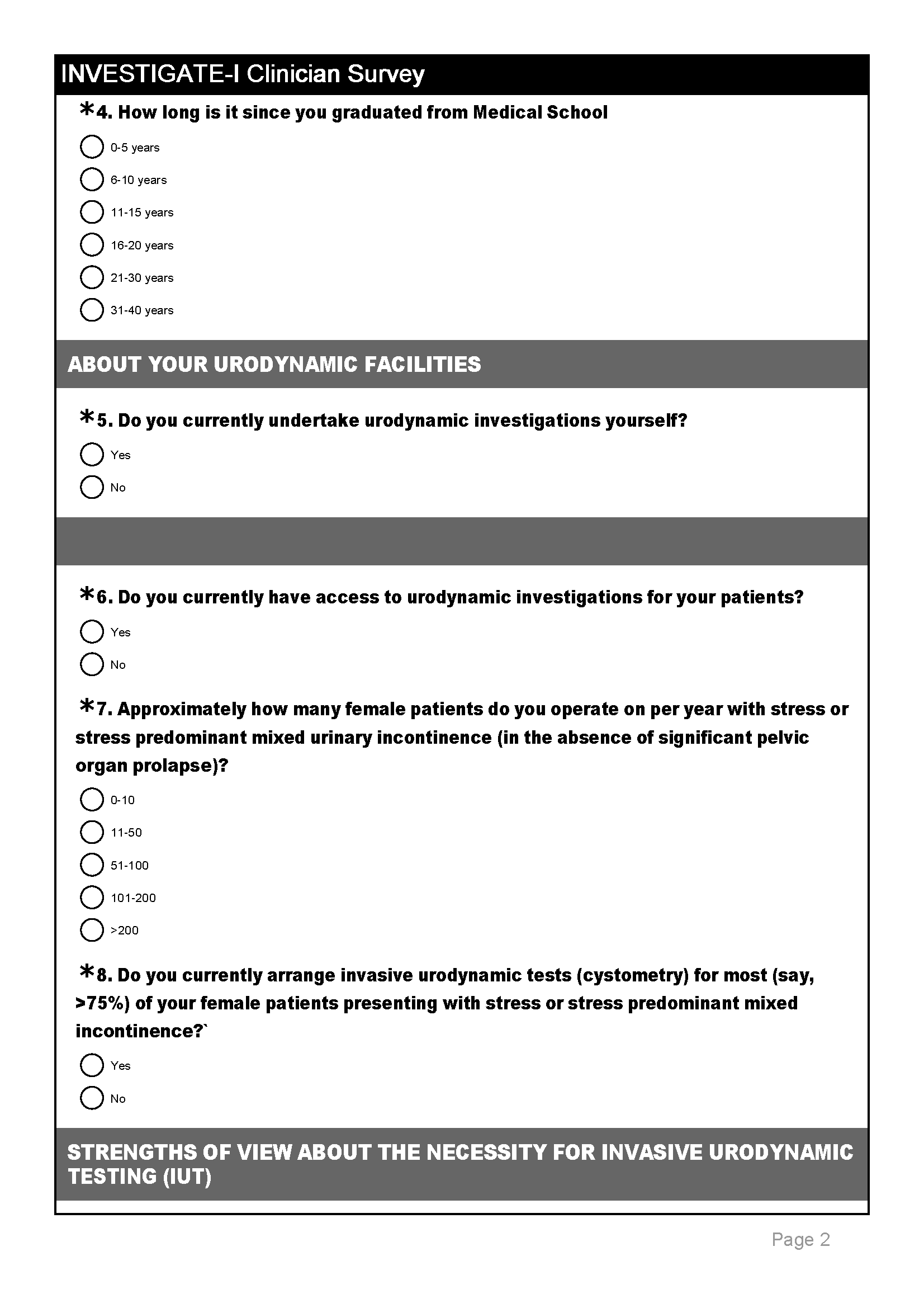

Supplement: Supplementary file 2 [file nau0031-1223-sd2.tif]

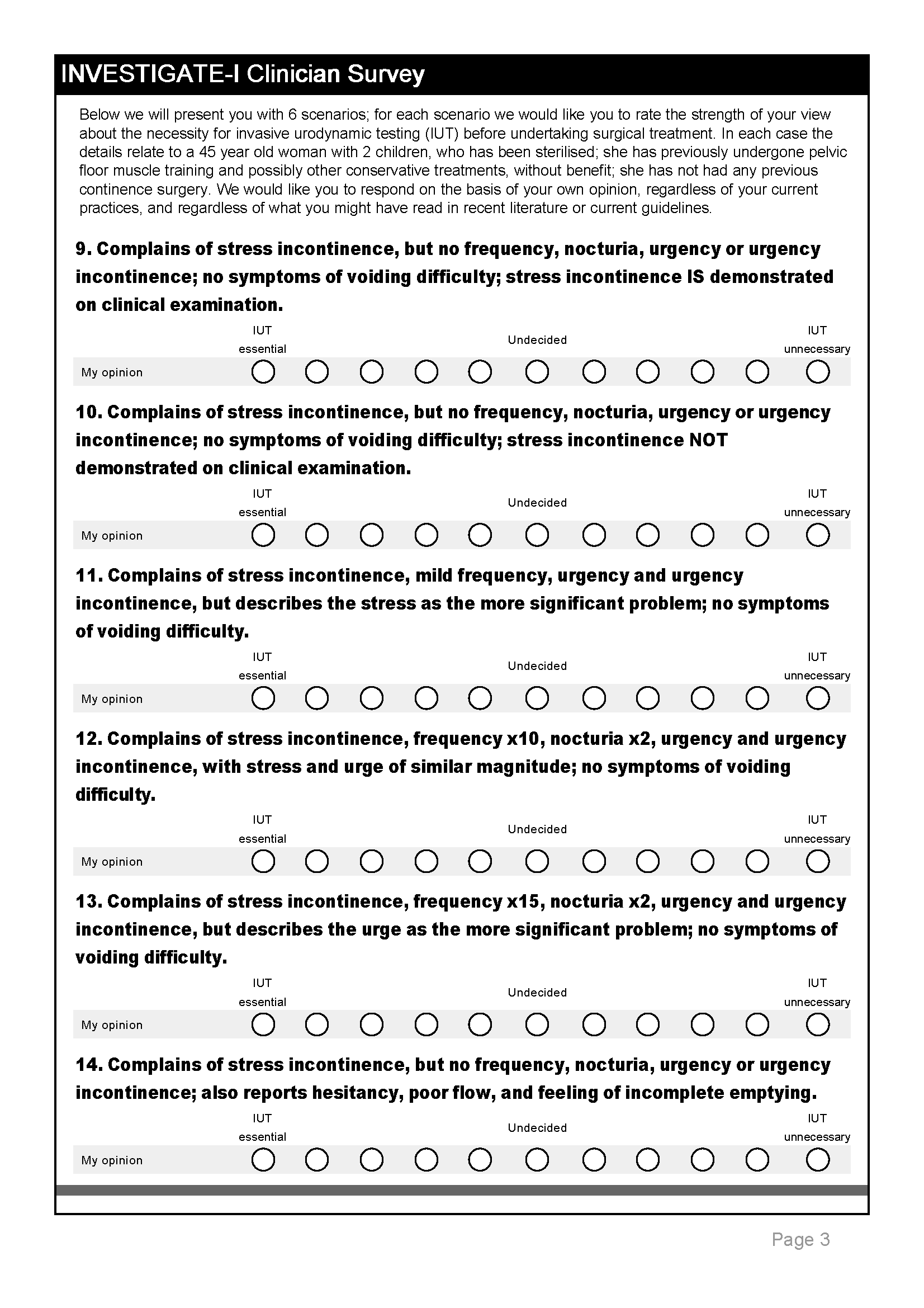

Supplement: Supplementary file 3 [file nau0031-1223-sd3.tif]

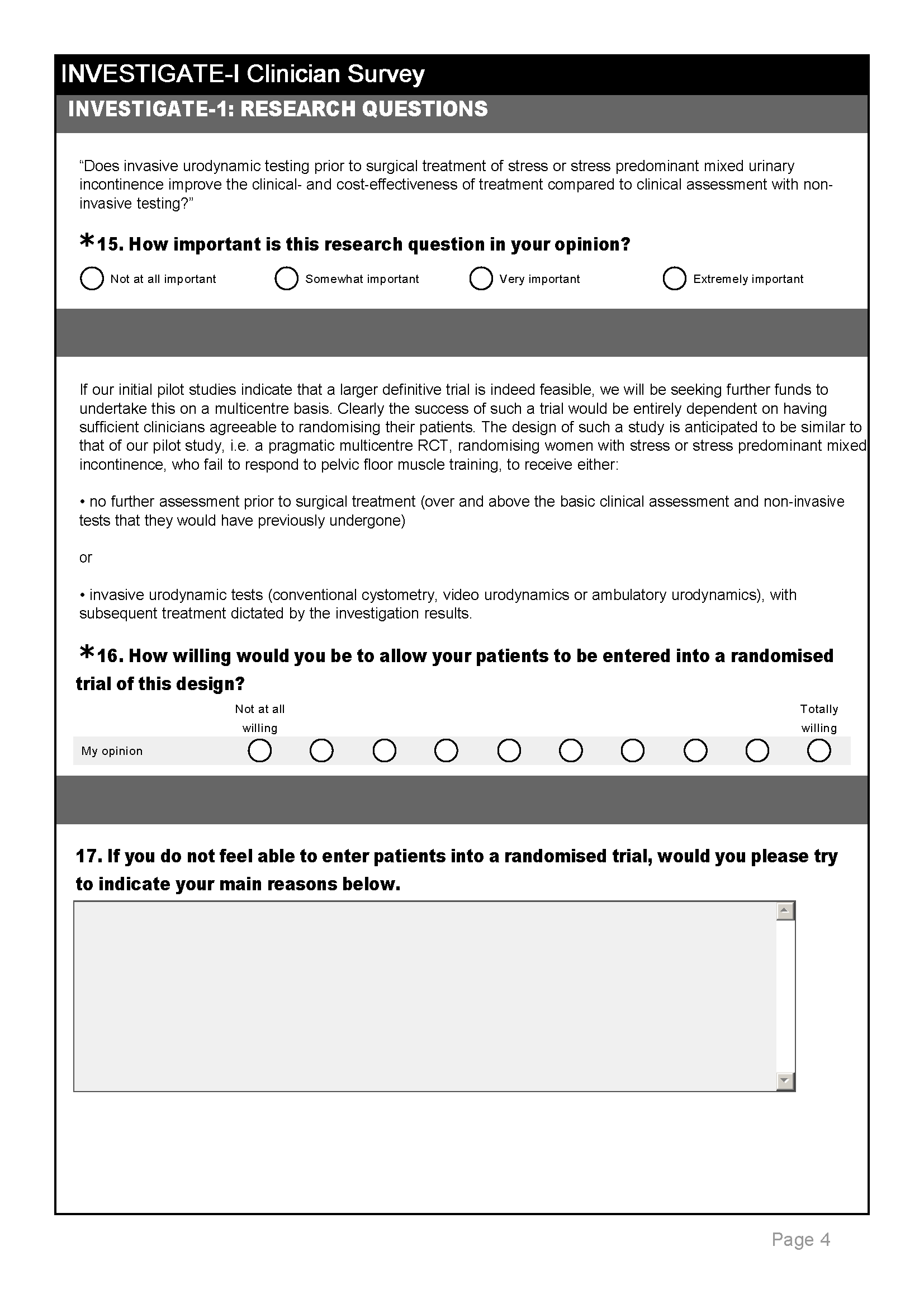

Supplement: Supplementary file 4 [file nau0031-1223-sd4.tif]

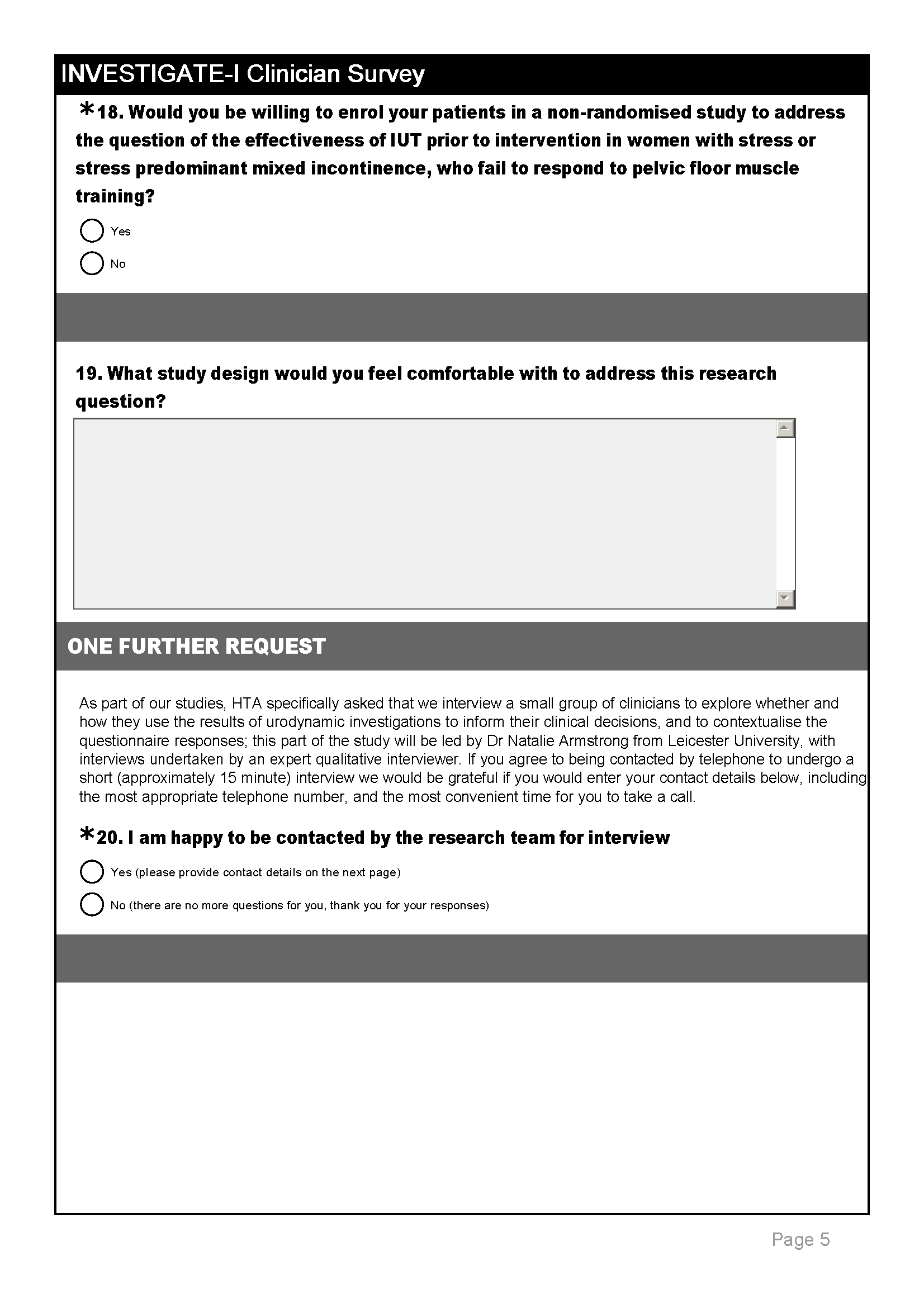

Supplement: Supplementary file 5 [file nau0031-1223-sd5.tif]

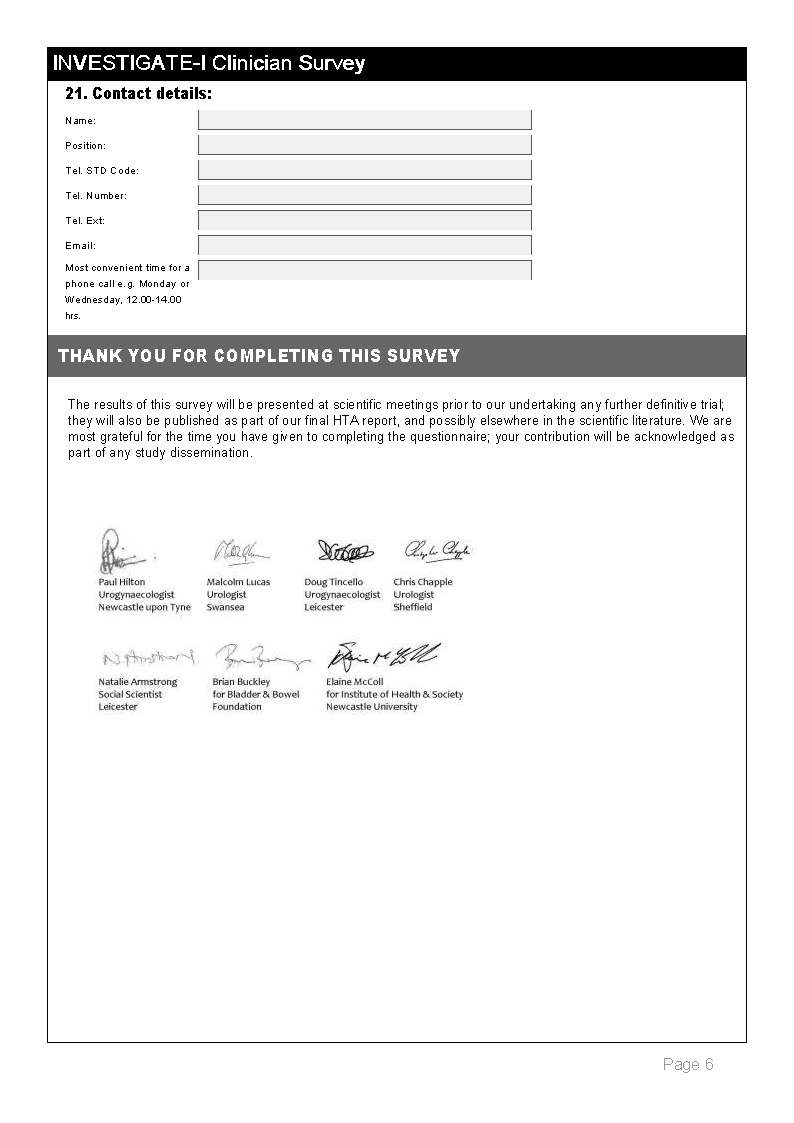

Supplement: Supplementary file 6 [file nau0031-1223-sd6.tif]
